# The association between self-efficacy and self-care in essential hypertension: a systematic review

**DOI:** 10.1186/s12875-021-01391-2

**Published:** 2021-02-22

**Authors:** Felicia Clara Jun Hui Tan, Prawira Oka, Hajira Dambha-Miller, Ngiap Chuan Tan

**Affiliations:** 1 Ministry of Health Holdings, 1 Maritime Square, #11-25 HarbourFront Centre, Singapore, 099253 Singapore; 2 SingHealth Polyclinics, 167 Jalan Bukit Merah Connection One (Tower 5), #15-10, Singapore, 150167 Singapore; 3 University of Southampton, Southampton, SO17 1BJ UK; 4 SingHealth-Duke NUS Family Medicine Academic Clinical Programme, Singapore, Singapore

**Keywords:** Self-efficacy, Self-care, Essential hypertension, Systematic review

## Abstract

**Background:**

The successful management of hypertension requires sustained engagement in self-care behaviour such as adhering to medication regimens and diet. Bandura’s Social Cognitive Theory suggests that self-efficacy is a major determinant of engagement in self-care behaviour. Self-efficacy refers to an individual’s belief in their capacity to execute behaviours necessary to produce specific performance attainments. This systematic review of observational studies aims to summarise and evaluate the quality of evidence available to support the association between self-efficacy and engagement in self-care behaviour in hypertension.

**Methods:**

Searches were performed of the Pubmed, MEDLINE, CINAHL and OpenSIGLE databases from database inception to January 2020. Reference lists and individual journals were also hand searched. Observational studies in English quantifying self-efficacy and self-care behaviour in hypertensive adults were included. The quality of included articles was assessed with the National Institute of Health Quality Assessment Tool for observational studies.

**Results:**

The literature search identified 102 studies, of which 22 met the inclusion criteria for full-text review. There were 21 studies which reported that higher self-efficacy was associated with engagement in self-care behaviours including medication adherence (*n* = 9), physical activity (*n* = 2) and dietary changes (*n* = 1). Of these, 12 studies were rated as ‘good’ on the quality assessment tool and 10 were ‘fair’. A common limitation in these studies was a lack of objectivity due to their reliance on self-reporting of engagement in self-care behaviour.

**Conclusion:**

Our review suggests an association between self-efficacy and self-care. However, the evidence supporting this association is of low to medium quality and is limited by heterogeneity. Our findings suggest the need for further well-designed interventional studies to investigate this association.

## Background

Essential hypertension is prevalent globally. Hypertension is diagnosed when an individual is found to have a clinic blood pressure of 140/90 mmHg (millimetres of mercury) or higher, or an ambulatory blood pressure daytime average or home blood pressure average of 135/85 mmHg (millimetres of mercury) or higher [1]. Hypertension increases the risk of cardiovascular events and is a major cause of premature death worldwide [2]. The World Health Organisation estimated that 1.13 billion people worldwide had hypertension in 2019, which has significantly increased from 594 million in 1975 [2]. The prevalence of hypertension ranges from 30.0 to 71.6% among adults and older individuals [3]. However, hypertension remains poorly controlled worldwide. In 2010, only 13.8% of adults worldwide had controlled hypertension [4]. Therapeutics alone are insufficient to optimise blood pressure control in these adults; they are also recommended to make lifestyle changes to better control their chronic disease [1].

Self-care encompasses the actions that individuals take to lead a healthy lifestyle, care for their chronic illness and to prevent further illness [5]. In hypertension, these self-care behaviours recommended for optimal disease control include: (a) adhering to anti-hypertensive medication, (b) adhering to a healthy diet low in salt, (c) engaging in adequate physical activity, (d) smoking cessation and (e) consuming alcohol in moderation [1]. In randomised controlled trials, dietary changes, exercise interventions and interventions to reduce alcohol consumption have been shown to produce a significant reduction in blood pressure [6].

Self-care adherence is low among adults with hypertension [7]. They are often unwilling to make the recommended behavioural changes [8]. The percentage of respondents reporting non-adherence to medications ranged from 24.1% (in the Netherlands) to 70.3% (in Hungary) in a cross-sectional study involving nine European countries performed in 2015. In England and Wales, 41.5 and 38.1% respectively of adults with hypertension reported non-adherence [9]. In the United States of America, a study done among African-Americans reported that only 52.2% of participants engaged in adequate physical activity, while 22.0% adhered to diet recommendations. Only slightly more than half (58.6%) the participants were adherent to their medication regimen [10].

One of the barriers to self-care was identified as a lack of motivation for behaviour change [11]. Self-efficacy may be a key to improving motivation and thereby, engagement in self-care behaviour in hypertension. Self-efficacy refers to “an individual’s belief in his or her capacity to execute behaviours necessary to produce specific performance attainments” [12]. Bandura’s Social Cognitive Theory suggests that self-efficacy influences motivation and the ability to engage in self-care behaviours [13]. According to this theory, personal cognitive and affective factors (such as belief and self-efficacy) and environmental factors (such as social support) contribute to a dynamic, ongoing process which influences self-care behaviour. Individuals with higher perceived self-efficacy are able to motivate themselves to engage regularly in self-care behaviour and overcome obstacles which prevent them from performing these behaviours, for example, a lack of time or desire to perform the behaviour [14]. They are more likely to start engaging in self-care behaviour and to maintain it over the long term [13]. In this way, enhanced self-efficacy is associated with improved health status in the areas affected by these self-care behaviours [15]. Lorig’s work shows that in the management of chronic disease such as arthritis, a higher self-efficacy is associated with improved health outcomes such as decreased pain and fatigue [16].

Engagement in self-care behaviour may also be hindered by the lack of symptoms in essential hypertension. In Lorig’s self-management programme, originally designed for arthritis and later generalised to chronic disease, self-care behaviour is intended to decrease symptoms such as pain and depression [17, 18]. Without symptoms to serve as a prompt, individuals may have less impetus to engage in self-care behaviour.

The relationship between self-efficacy and self-care is also compounded by the variety of behavioural changes required for the optimal management of essential hypertension. Self-efficacy is task-specific [13]; self-efficacy on one task may not influence self-efficacy on another. Additionally, each self-care behaviour has its barriers to regular performance. For example, the barriers to medication adherence (concerns about side effects, costs of medications [19]) are different from the barriers to smoking cessation (anxiety, easy access to cigarettes [20]).

Thus, this systematic review aims to summarise and evaluate the quality of evidence available to support the association between self-efficacy and engagement in self-care behaviour in hypertension.

## Method

A protocol detailing the search methods employed was registered on PROSPERO (registration number CRD42020171290).

### Data sources

Searches were carried out on the following databases: PubMed, MEDLINE and CINAHL, and the grey literature database OpenSIGLE. The databases were searched from database inception to January 2020. Terms were combined using Boolean logic commands: (“hypertension” [ti] AND (“self-efficacy” [ti] OR “self-efficacy” [ab])).

The journals were hand-searched from January 2010 or its inception (whichever was later) to the latest issue as of January 2020. The reference lists of the selected articles were also hand-searched. The literature search process as described was performed by two authors (Felicia Clara Tan and Prawira Oka) independently. The results from each author were compared; no discrepancies were identified.

### Study selection and quality appraisal

The inclusion criteria for the articles are:

(a) full-text observational studies published in English.

(b) involve adult participants aged 18 years and over with essential hypertension.

(c) measure the relationship between participants’ level of self-efficacy and their performance of one or more of the following self-care behaviours: (a) adhering to anti-hypertensive medication, (b) adhering to a healthy diet low in salt (c) engaging in adequate physical activity, (d) smoking cessation and (e) consuming alcohol in moderation. Other types of research studies and those not related to essential hypertension nor any of the specified self-care behaviour and self-efficacy were excluded.

**Fig. 1:**
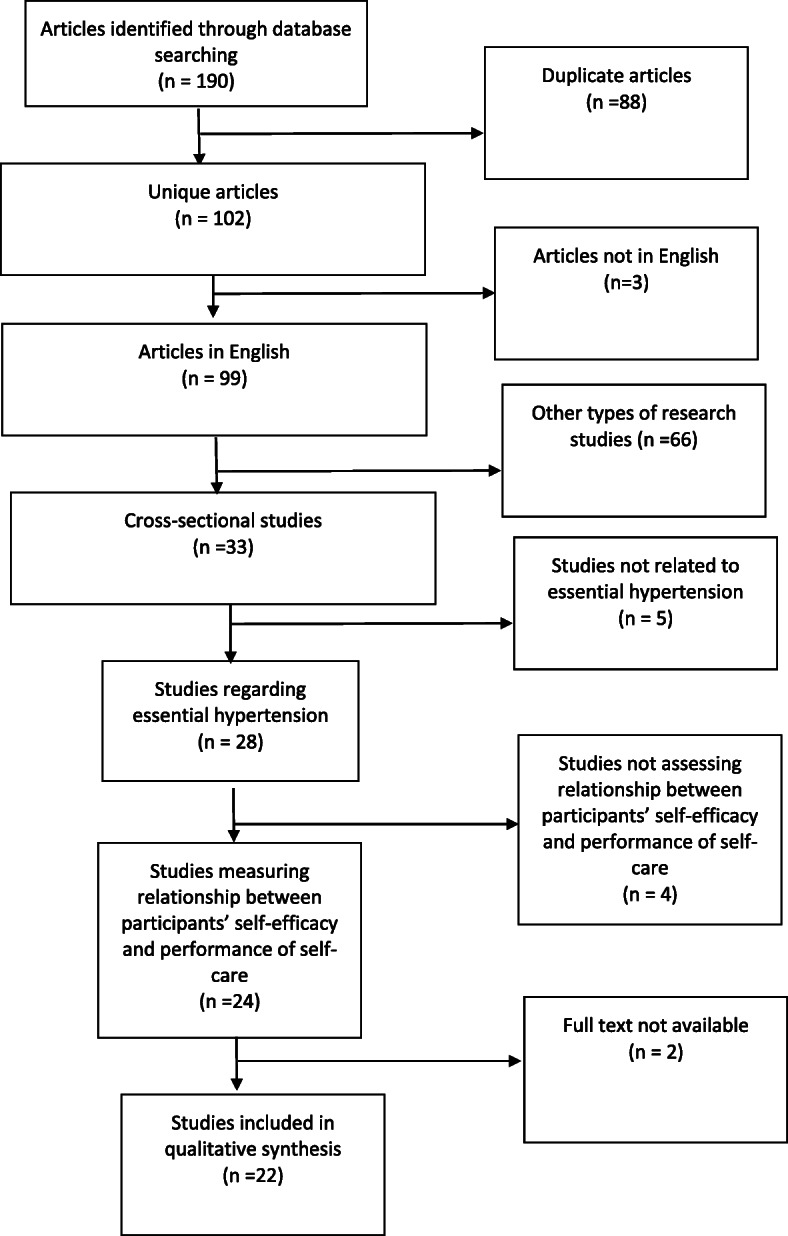
Process of article selection. Legend: Unique articles were identified from database searching. Articles were screened against eligibility criteria by two independent authors. Articles for which the full text was unavailable were excluded from review

Two authors (Felicia Clara Tan and Prawira Oka) screened articles against inclusion and exclusion criteria independently, with disagreements resolved by an independent arbitrator (Ngiap Chuan Tan). In screening each article against the inclusion and exclusion criteria, the full text of each article was reviewed by each author independently. A total of 80 papers, including sub-studies, were rejected for failing to meet all the inclusion criteria. A total of 22 articles were eventually examined in this review. The process of article selection is detailed in Fig. 1.

To evaluate the quality of these articles, each article was graded using the National Institute of Health (NIH) Quality Assessment Tool for Observational Cohort and Cross-Sectional Studies by two independent authors (Felicia Clara Tan and Prawira Oka) [21]. Their results were compared and discrepancies identified and resolved by an independent arbitrator (Ngiap Chuan Tan). The outcome of the grading is presented in Table 1.

**Table 1:** Cross-sectional studies graded by NIH Quality Assessment Tool [21]

No.	Name of paper	Q1	Q2	Q3	Q4	Q5	Q6	Q7	Q8	Q9	Q10	Q11	Q12	Q13	Q14	QR
1	Ahn & Ham 2016 [22]	Y	N	NR	NR	Y	N	N	Y	CD	N	Y	N	NA	Y	Fair
2	Al Noumani 2018 [23]	Y	Y	NR	Y	Y	N	N	Y	Y	N	Y	N	NA	Y	Good
3	Bae et al. 2016 [24]	Y	Y	NR	Y	N	N	N	Y	Y	N	N	N	NA	Y	Fair
4	Bahari et al. 2019 [25]	Y	Y	N	Y	Y	N	N	Y	Y	N	Y	N	NA	Y	Good
5	Breaux-Shropshire et al. 2012 [26]	Y	N	Y	NR	N	N	N	Y	Y	N	Y	N	NA	Y	Fair
6	Chang & Sok, 2015 [27]	Y	Y	Y	Y	Y	N	N	Y	Y	N	Y	N	NA	Y	Good
7	Ea et al., 2018 [28]	Y	Y	NR	Y	Y	N	N	Y	Y	N	Y	N	NA	Y	Good
8	Elder et al., 2012 [29]	Y	Y	NR	Y	N	N	N	Y	Y	N	Y	N	NA	Y	Fair
9	Gacek, 2014 [30]	Y	Y	NR	Y	N	N	N	Y	NR	N	NR	N	NA	N	Fair
10	Giena, Thongpat & Nitirat, 2018 [31]	Y	Y	NR	Y	N	N	N	Y	Y	N	Y	N	NA	Y	Fair
11	Heydari et al. 2014 [32]	Y	Y	NR	Y	N	N	N	Y	Y	N	Y	N	NA	N	Fair
12	Hu, Li & Arao, 2015 [33]	Y	Y	Y	Y	N	N	N	Y	Y	N	Y	N	NA	Y	Good
13	Idowu et al., 2012 [34]	Y	Y	Y	Y	N	N	N	Y	Y	N	Y	N	NA	Y	Good
14	Khalesi, Irwin & Sun, 2017 [35]	Y	Y	NR	Y	Y	N	N	Y	Y	N	Y	N	NA	Y	Good
15	Lee & Park, 2017 [36]	Y	Y	Y	Y	Y	N	N	Y	Y	N	Y	N	NA	Y	Good
16	Lee et al., 2010 [37]	Y	Y	NR	Y	N	N	N	Y	Y	N	Y	N	NA	Y	Fair
17	Ma, 2018 [38]	Y	Y	Y	Y	Y	N	N	Y	Y	N	Y	N	NA	Y	Good
18	Nafradi et al. 2016 [39]	Y	N	NR	NR	N	N	N	Y	Y	N	Y	N	NA	N	Fair
19	Namwong et al. 2015 [40]	Y	Y	NR	Y	Y	N	N	Y	Y	N	Y	N	NA	Y	Good
20	Pinprapapan et al., 2013 [41]	Y	Y	NR	Y	Y	N	N	Y	Y	N	Y	N	NA	Y	Good
21	Son & Won 2017 [42]	Y	Y	NR	Y	Y	N	N	Y	Y	N	Y	N	NA	Y	Good
22	Warren-Findlow et al. 2012 [43]	Y	Y	NR	Y	N	N	N	N	Y	N	Y	N	NA	Y	Fair

Each study was assessed for quality with the NIH Quality Assessment Tool by two independent authors. Differences in grading were resolved by an arbitrator. The final grading of each article on each question in the NIH Quality Assessment Tool is shown here

*Y* Yes, *N* No, *CD* Cannot determine, *NR* Not reported, *NA* Not applicable, *QR* Quality Rating

Q1: 1. Was the research question or objective in this paper clearly stated?

Q2: 2. Was the study population clearly specified and defined

Q3: 3. Was the participation rate of eligible persons at least 50%?

Q4: 4. Were all the subjects selected or recruited from the same or similar populations (including the same time period)? Were inclusion and exclusion criteria for being in the study pre-specified and applied uniformly to all participants?

Q5: 5. Was a sample size justification, power description, or variance and effect estimates provided?

Q6: 6. For the analyses in this paper, were the exposure(s) of interest measured prior to the outcome(s) being measured?

Q7: 7. Was the timeframe sufficient so that one could reasonably expect to see an association between exposure and outcome if it existed?

Q8: 8. For exposures that can vary in amount or level, did the study examine different levels of the exposure as related to the outcome (e.g., categories of exposure, or exposure measured as continuous variable)?

Q9: 9. Were the exposure measures (independent variables) clearly defined, valid, reliable, and implemented consistently across all study participants?

Q10: 10. Was the exposure(s) assessed more than once over time?

Q11: 11. Were the outcome measures (dependent variables) clearly defined, valid, reliable, and implemented consistently across all study participants?

Q12: 12. Were the outcome assessors blinded to the exposure status of participants?

Q13: 13. Was loss to follow-up after baseline 20% or less?

Q14: 14. Were key potential confounding variables measured and adjusted statistically for their impact on the relationship between exposure(s) and outcome(s)?

### Data synthesis

The full text of each selected article was reviewed to extract key information for summarising into a table. This included the design of the study, population sampled, instruments used, outcomes measured (including which domains of self-care behaviour were studied) and the main conclusions (Table 2). Key findings from the included studies were narratively synthesised.

**Table 2:** Summary of cross-sectional studies selected for analysis

No	Population	Intervention	Outcome	
1	Ahn & Ham, 2016 [22] **Population:** 289 adults receiving medical aid across South Korea, recruited from the community	**Instrument:** General self-efficacy – measured by 17 questions [44] translated into Korean [45] **Comparison made:** Independent variable: Self-efficacy; dependent variable: medication adherence	Medication adherence – Modified Morisky Scale [46]	In hierarchical multiple regression analysis, self-efficacy was significantly associated with medication adherence (step 2 β = 0.143, step 3 β = 0.146, *p* = 0.019)
2	Al-Noumani et al. 2018 [23] **Population**: 215 Omanis aged 21 years or older from primary healthcare settings around Oman	**Instrument used:** Self-efficacy for medication adherence – Medication Adherence Self-Efficacy Scale- Revised (MASES-R) translated into Arabic **Comparison made:** Independent variable: self-efficacy for medication adherence, dependent variable: medication adherence	Self-care behaviours assessed: Medication adherence – Morisky Medication Adherence Scale-8 items translated into Arabic	Participants with higher self-efficacy were 2.5 times more likely to have high medication adherence (OR = 2.59, *p* < 0.01)
3	Bae et al. 2016 [24] **Population:** 401 rural community-dwelling elderly (aged 65 and over) in South Korea, recruited from the community	**Instrument:** Medication adherence self-efficacy scale – revision (MASES-R) [47] **Comparison made:** Independent variable: Self-efficacy for medication adherence; dependent variable: medication non-adherence	Medication adherence – 6 yes or no questions to assess intentional and unintentional non-adherence [48]	Self-efficacy has a significant direct influence on unintentional nonadherence behaviours (β = −0.433, *P* < 0.001), significant indirect effect on unintentional adherence (β = −0.286, P < 0.001) but no significant direct effect on intentional non-adherence (β = − 0.055, *P* = 0.515)
4	Bahari et al., 2019 [25] **Population:** 158 Saudi men aged 18 and above, attending primary health care centres in the Jizan and Al-Sharqia regions of Saudi Arabia	**Instrument used:** General self-efficacy - Hypertension Self-Care Profile [49] translated into Arabic **Comparison made:** Independent variable: Self-efficacy; dependent variable: performance of self-care behaviours	Self-care behaviours assessed: physical activity, adherence to diet, abstention from alcohol and smoking, self-monitoring of BP, weight control, regular doctor visits, stress reduction [49] - Hypertension Self-Care Profile translated into Arabic	Self-efficacy is significantly associated with performance of self-care behaviours (β = 0.353, *p* < 0.05) Self-efficacy fully mediates the relationship between family social support and hypertension self-care behaviours
5	Breaux-Shropshire et al., 2012 [26] **Population**: 149 municipal employees with access to health insurance, recruited from participants of an employee wellness programme in the United States of America	**Instrument:** Medication adherence self-efficacy – revised Medication Adherence Self-Efficacy Scale (MASES-R) [47] **Comparison made**: Independent variable: Self-efficacy for medication adherence; dependent variable: medication adherence	Medication adherence – 8 item Morisky Medication Adherence Scale [50]	Significant positive linear relationship between medication adherence and medication adherence self-efficacy (r = 0.549, *p* < 0.05)
6	Chang & Sok, 2015 [27] **Population:** 306 Koreans aged 65 and above, recruited from public health centres in Seoul, Korea	**Instrument used:** SE for physical activity – Korean translation of the instrument “Exercise Self-efficacy Measure” [51, 52] **Comparison made:** Independent variable: Self-efficacy; dependent variable: Sedentary behaviour and performance of physical activity	Sedentary behaviour and physical activity - Korean translation of International Physical Activity Questionnaire-Short Form (IPAQ-SF) [53, 54]	Self-efficacy was not one of the predictors of sedentary behaviour. Sedentary behaviour was instead significantly predicted by variables such as empowerment level, perceived health, time since diagnosis of hypertension, vigorous-intensity physical activity, and depression, which explained 42.6% of the variance in sedentary behaviour*.*
7	Ea et al., 2018 [28] **Population:** 163 adult (aged at least 18) first-generation Filipino immigrants in the United States, recruited from the community	**Instrument used:** Self-efficacy – Hypertension self-care profile self-efficacy scale [49] **Comparison made:** Independent variable: Self-efficacy to engage in various aspects of hypertension management, dependent variable: tendency to engage in hypertension self-care behaviours	Self-care behaviours assessed: adherence to appropriate diet, adherence to medications, smoking cessation, regular exercise, stress avoidance and use of relaxation techniques - Medical Outcomes Study Specific Adherence Scale [55]	Self-efficacy is positively correlated with hypertension self-care (correlation coefficient = 0.407, *p* < 0.001), self-efficacy is a significant predictor of hypertension self-care (β = 0.270, *p* = 0.003)
8	Elder et al., 2012 [29] **Population:** 235 Southern African American men aged 18 or over, recruited from a hospital in Alabama, USA	**Instrument used:** Self-efficacy - Ogedegbe Self-Efficacy Scale [56] **Comparison made:** Independent variable: Self-efficacy; dependent variables: Medication adherence	Medication adherence – measured by Morisky Medication Adherence Scale [50]	Participants with higher self-efficacy more likely to report better medication adherence (OR = 1.08; 95% CI = 1.02)
9	Gacek, 2014 [30] **Population:** 160 women from Małopolska, Poland, aged 45–60	**Instrument used:** General self-efficacy – General Self-Efficacy Scale (35, as cited in Gacek, 2014) **Comparison made:** Independent variable: Self-efficacy; dependent variable: Adherence to recommended diet	Frequency of consumption of food products – measured using a seven-item scale	Higher levels of self-efficacy were associated with more frequent consumption of recommended food products
10	Giena, Thongpat & Nitirat, 2018 [31] **Population**: 333 adults aged 60 and above from 4 primary health centres in Bengkulu City, Indonesia	**Instrument used:** Self-efficacy – Self-rated Abilities for Health Practice Scale (54, as cited in Giena et al. 2017) **Comparison made:** Independent variable: Self-efficacy; dependent variable: Performance of self-care behaviour	Self-care - Measured by modified version of Health Promoting Lifestyles Profile II (55, as cited in Giena et al. 2017)	Self-efficacy (among other factors) significantly affects health-promoting behaviour (β = 0.321. *P* < 0.001)
11	Heydari et al., 2014 [32] **Population:** 671 adults with hypertension aged 30 and above referred to rural health care centres in Ardabil city, Iran in 2013	**Instrument:** Health belief model questionnaire, which included 6 items on self-efficacy [32] **Comparison made:** Independent variable: self-efficacy; dependent variable: medication adherence	Medication adherence – Morisky Medication Adherence Scale-4	Individuals with moderate self-efficacy more likely to be non-adherent than adherent to medication (OR 1.5, P < 0.001), individuals with low self-efficacy more likely to be non-adherent than adherent to medication (OR 5.1, *p* < 0.001)
12	Hu, Li & Arao, 2015 [33] **Population:** 318 residents of a rural community in Beijing aged 35 and above, recruited from the community	**Instruments used:** Self-efficacy - validated Chinese version of the Self-Efficacy for Managing Chronic Disease six-Item Scale [57] **Comparison made:** Independent variable: Self-efficacy; dependent variable: Performance of self-care behaviour	Self-care behaviours assessed: medication adherence, regular BP measurement, physical exercise, alcohol abstinence, smoking cessation & low salt diet adherence - assessed using face-to-face questionnaires	Higher self-efficacy is associated with engagement in exercise. A 10-unit increase in self-efficacy is related to an increased odds ratio of 1.25 (95% CI 1.04–1.49) for performing regular exercise.
13	Idowu et al., 2013 [34] **Population:** ﻿212 adults aged 31 and above receiving treatment from two tertiary health centres in Nigeria	**Instruments used:**- Self-efficacy for exercise - Exercise Self-Efficacy Scale (43, as cited in Idowu et al., 2012) **Comparison made:** Independent variable: Self-efficacy; dependent variable: Engagement in physical activity	Physical activity level - International Physical Activity Questionnaire [58]	Significant associations between physical activity levels and self-efficacy (rs = 0.67, *p* < 0.01)
14	Khalesi, Irwin & Sun, 2017 [35] **Population:** 270 adults aged 18 and over in Gold Coast, Australia, recruited from the community	**Instrument used:** Self-efficacy for diet and exercise- short version of self-efficacy questionnaire developed by Sallis et al. [59] **Comparison made:** Independent variable: Self-efficacy for exercise; dependent variable: medication adherence	Self-care behaviours assessed: Adherence to recommended diet – Food Frequency Questionnaire [60], Adherence to medication – 4 questions, modified and validated for purposes of this study, containing 4 items on medication, compliance and reasons for non-compliance [61]	Exercise self-efficacy was associated with a higher likelihood of good adherence to antihypertensive medication (t = 2.38, *p* = 0.01), self-efficacy for adherence to diet not significantly associated with good adherence to antihypertensive medication (t = 1.13, *p* = 0.25)
15	Lee & Park, 2017 [36] **Population:** 255 adults aged 65 and over attending hospitals in Kyung-buk province of South Korea	**Instrument:** SE- measured with 10-item questionnaire with scale from 10 to 100 (43, as cited in Lee & Park, 2017) **Comparison made:** Independent variable: self-efficacy; dependent variable: engagement in self-care behaviour	Self-care behaviour – 16 item questionnaire including items regarding management of diet, body weight, alcohol, smoking, stress, coffee, medication and exercise [62]	In participants with controlled hypertension, self-efficacy affected SC behaviour (β = 15.41, *p* = .009), in participants with uncontrolled hypertension, self-efficacy was the strongest factor affecting self-care behaviour (β = 0.45, *p* < .001) among the factors analysed
16	Lee et al., 2010 [37] **Population:** 445 middle-aged (40–64 years) Korean Americans from the community	**Instruments used:** Hypertension control self-efficacy - Self-efficacy Scale, modified instrument based on the Hypertension Belief Scale [63] **Comparison made:** Independent variable: Self-efficacy; dependent variable: Performance of self-care behaviour	Self-care behaviours – measured by 5 items in questionnaire (medication adherence, healthy diet, weight control, & exercise).	Self-efficacy positively associated with performance of self-care behaviour (β = 0.246, *p* < 0.001)
17	Ma, 2018 [38] **Population:** 382 adults aged between 18 and 59 attending two tertiary hospitals in Guangzhou, China	**Instrument used:** Health belief questionnaire for hypertensive patients – 29 items grouped in five dimensions of health beliefs, of which self-efficacy was one (8 items in the questionnaire measured self-efficacy**)** **Comparison made:** Independent variable: Self-efficacy; dependent variable: engagement in self-care behaviours	Hypertension self-care behaviours assessed: BP monitoring, medication, dietary, physical activity, weight management, smoking and alcohol management – Hypertension self-care behaviours questionnaire (11, as cited in Ma, 2018)	Self-efficacy is the strongest determinant of self-care behaviours (β = 0.62, p < 0.001)
18	Nafradi et al. 2016 [39] **Population:** 109 adults with hypertension aged over 35 years, recruited from medical offices and hospitals	**Instrument:** Self-efficacy – Medication Adherence Self-Efficacy Scale (MASES) [47] **Comparison made:** Independent variable: Self-efficacy for medication adherence; dependent variable: medication non-adherence	Medication adherence – 15 item scale developed based on the Medication Adherence Report Scale [64] (as cited in Nafradi et al. 2016) – included two separate sub-scales for intentional and unintentional non-adherence	Lower adherence self-efficacy is a determinant of intentional non-adherence (t = 4.54, p < 0.001) and unintentional non-adherence (t = 3.15, *p* = 0.002
19	Namwong et al., 2015 [40] **Population:** 341 Thais aged 60 and above attending hypertension clinics in a community hospital in northern Thailand	**Instrument:** Self-efficacy – Hypertensive Self-efficacy Scale [41] **Comparison made:** Independent variable: self-efficacy for medication adherence, adherence to diet, weight control, physical exercise; dependent variable: medication adherence	Self-care– adherence to medications, adherence to diet, weight control, smoking cessation, adherence to exercise, limiting alcohol intake, stress management and four attributes of adherence i.e. (i) alignment of individuals’ behaviours and health recommendations (ii) mastery of new behaviours and health knowledge (iii) ongoing collaboration with health care providers on treatment plan (iv) individuals’ perceived ability to meet optimal blood pressure - Hypertensive Adherence to Therapeutic Regimens Scale [41]	Perceived self-efficacy had a significant direct effect on medication adherence (structural path coefficient 0.69, *p* < 0.01)
20	Pinprapapan et al., 2013 [41] **Population:** 321 adults in Northern Thailand aged 35–59 recruited from a community hospital	**Instruments used:** Self-efficacy for managing hypertension –Hypertensive Self-efficacy Scale **Comparison made:** Independent variable: Self-efficacy; dependent variables: Performance of self-care behaviours	Self-care behaviours – adherence to medications, adherence to diet, weight control, smoking cessation, adherence to exercise, limiting alcohol intake, stress management and four attributes of adherence i.e. (i) alignment of individuals’ behaviours and health recommendations (ii) mastery of new behaviours and health knowledge (iii) ongoing collaboration with health care providers on treatment plan (iv) individuals’ perceived ability to meet optimal blood pressure - Hypertensive Adherence to Therapeutic Regimens Scale	Direct positive influence of perceived self-efficacy on performance of self-care behaviours (structural path coefficient = 0.54, p < 0.01)
21	Son & Won 2017 [42] **Population:** 255 adults aged 65 and over at a general hospital in Seoul, Korea	**Instrument:** Self-efficacy for medication adherence – Korean version of self-efficacy for appropriate medication use scale [65] **Comparison made:** Independent variable: Self-efficacy for medication adherence; dependent variable: medication adherence	Medication adherence – Korean version of 8-item MMAS-B [66]	Self-efficacy is significantly positively correlated with medication adherence (r = 0.53, p < 0.001), self-efficacy is significantly predictive of medication adherence (β = .55, *P* < .001)
22	Warren-Findlow et al. 2012 [43] **Population:** 190 African-Americans aged 21 years and above, recruited from the community in the greater metropolitan Charlotte area, USA	**Instrument:** Self-efficacy - five-item scale modified from existing validated measure to assess self-efficacy to manage disease [67] **Comparison made:** Independent variable: self-efficacy; dependent variables: engagement in various self-care activities	Self-care activities –medication adherence, adherence to low-salt diet, engagement in physical activity, practising weight management techniques, not smoking - measured by Hypertension Self-Care Activity Level Effects [10] alcohol intake – measured by NIAAA Quantity and Frequency Questionnaire [68] (as cited in Warren-Findlow et al. 2012)	Good self-efficacy statistically significantly associated with increased prevalence of adherence to medication (Prevalence ratio (PR) = 1.23), eating a low salt diet (PR = 1.64), engaging in physical activity (PR = 1.27), not smoking (PR = 1.10), practising weight management techniques (PR = 1.63)

Summary of population studied, instruments used, comparison made and outcome reported in each article that was selected for review

## Results

Searches in Pubmed, MEDLINE, CINAHL and OpenSIGLE yielded 86, 50, 49 and zero articles in English respectively. Hand searching of journals yielded one article while hand searching of the reference lists of selected articles yielded four articles. After eliminating duplicates, a combined total of 102 articles were identified. There were 22 articles which met all the eligibility criteria for inclusion in this review and were examined in full text. Key information from each article is summarised in Table 2.

Of the 22 cross-sectional studies included, ten were performed in Asia [22, 24, 27, 31, 33, 36, 37, 40–42], three in the Middle East [23, 25, 32], five in the United States of America [26, 28, 29, 37, 43], two in Europe [30, 39], one in Africa [34] and one in Australia [69]. All the studies involved adults, of which four [24, 36, 40, 42] involved exclusively elderly adults aged 60 or older. Of the 22 studies, 13 studies [23, 25, 27, 29, 31, 32, 34, 36, 38–42] involved participants recruited from healthcare settings while eight [22, 24, 26, 28, 33, 37, 43, 69] involved participants recruited from the community.

The 22 studies used a variety of instruments to measure their outcomes and studied samples from a variety of populations. Of the 22 studies, 21 reported that higher self-efficacy was associated with engagement in self-care behaviours. The remaining study [27] found no association between self-efficacy and engagement in self-care behaviours.

There were nine studies which reported an association between self-efficacy and general self-care behaviour [25, 28, 31, 36–38, 40, 41, 43], while 12 studies reported an association between specific self-care behaviours and self-efficacy for those behaviours. Among these 12 studies, nine reported an association between self-efficacy and medication adherence [22–24, 26, 29, 32, 35, 39, 42], two reported an association between self-efficacy and physical activity [33, 34] and one reported an association between self-efficacy and adherence to recommended diet [30].

There were two studies which reported no association between self-efficacy and specific self-care behaviours [27]. One [27] found that self-efficacy did not predict whether a person avoided being sedentary; the other [35] reported no significant association between self-efficacy for adherence to recommended diet and adherence to medication.

### Quality of included articles

All the included articles were judged to be of at least fair overall quality. All of them had a clearly stated research question. Most of them clearly specified and defined their study population, ensured participants were recruited from the same or similar populations, examined different levels of exposure, used clearly defined, valid and reliable exposure and outcome measures and adjusted for potential confounding variables. The most common flaws among the articles were a failure to obtain a high participation rate among eligible individuals and the lack of blinding. Additionally, none of the articles measured the association between self-efficacy and engagement in self-care behaviour over a period of time, as they were all cross-sectional studies.

The studies by Nafradi et al. and Idowu et al. were originally judged to be “poor” and “fair” by one of the reviewers respectively. The quality of these two studies were judged by the second reviewer to be “fair” and “good” respectively. The difference in judgement of quality for the study by Nafradi et al. was due to a difference in opinion regarding blinding and the validity of the instrument measuring the outcome. The difference in judgement of quality for the study by Idowu et al. was due to a difference in opinion regarding the validity of the instruments used to measure the exposure and outcome. After arbitration, the final judgement of the quality of these two studies by Nafradi et al. and Idowu et al. are “fair” and “good” respectively.

## Discussion

### Summary

This review reveals a low to moderate level of association between self-efficacy and self-care behaviours in hypertension. The implication for clinical practice is that adults with high self-efficacy are more likely to adhere to self-care in their management of hypertension.

### Strengths

This is a comprehensive and thorough synthesis of the available literature evaluating the association between self-efficacy and the performance of self-care behaviours in hypertension. It involved extensive database and hand searching that includes grey literature. It includes recently-published studies (from 2010 to 2019) which employ a variety of instruments and study a variety of populations. Thus, it is useful as a comprehensive summary of key findings regarding self-efficacy and self-care over the last decade. This summary may aid in planning interventions which aim to improve self-care through improving self-efficacy. By highlighting areas where further investigation is required, it is also useful in directing future research.

### Limitations

We have excluded articles not published in English. Authors who did not find positive results may have published their work in smaller, local journals not in English. Such articles could have been omitted from review because of the restriction to English language articles. Thus, there is a risk of introducing bias by including only English language articles. Certain databases e.g. EMBASE were not accessible because of limitations on institutional access, thus articles published only in those databases could have been omitted from review.

All the studies included were cross-sectional, which do not have the dimension of time. Observational studies examine observations but are unable to establish causative relationships. Overall, the level of evidence in observational studies is lower compared to other study designs, for example, a randomised controlled trial [70, 71]. Nonetheless, they are easy to conduct and often provide early data on the association to design subsequent adequately-powered controlled trials.

The results also provide no data on the relationship between self-efficacy and engagement in self-care over the long term. None of the studies included followed up on participants after data collection. Further research is needed to determine the sustained association between self-efficacy and self-care behaviour as hypertension is a chronic disease.

The included studies relied on self-reporting of self-care behaviour, which can be affected by recall bias. The level and types of engagement in self-care behaviour may fluctuate from day to day, which poses further challenges in its combined measurements using conventional questionnaires and tracking devices. A major limitation of the included studies was their failure to assess engagement in self-care behaviour using objective measurements. The instruments involved participants self-reporting their self-care behaviour without a record of objective measurements, such as calorie expenditure or minutes of physical exercise performed. This further increases the risk of recall bias.

Many of the included studies were also limited by flaws in their methodology. Most of them employed convenience sampling or failed to report their participation rate of eligible persons. Therefore, it is uncertain whether the samples they studied were representative of the population of interest. All the studies did not report that their assessors were blinded to the exposure status of their participants. The lack of blinding could have increased the risk of bias.

Most studies did not show an incremental association between self-efficacy and self-care behaviour. In six [25, 28, 31, 37, 40, 41] studies, the scores reflecting each self-care behaviour were simply added up and analysed as a total score. The optimum level of self-efficacy remains unclear for improved process or clinical outcomes in essential hypertension.

Meta-analysis could not be performed as the data was highly heterogeneous, with many different instruments used to measure self-efficacy and the performance of self-care. Better standardisation of the instruments used to measure self-efficacy and self-care and greater consensus on the instruments used may allow for quantitative analysis to be performed in future reviews.

### Comparison with existing literature

The finding that self-efficacy is correlated with self-care is consistent with Bandura’s proposition in the Social Cognitive Theory that self-efficacy drives self-care behaviour [13]. Individuals with low self-efficacy are less likely to engage in self-care behaviour. This is consistent with Bandura’s suggestion that self-efficacy influences behaviour by influencing individuals’ motivation for behavioural change [14]. Individuals with low self-efficacy have low motivation to change their behaviour. It is also consistent with previous studies investigating self-care behaviours in adults with hypertension which reported that self-care performance is low because individuals lack the motivation to change their lifestyles [8].

### Implications for practice and research

Individuals with essential hypertension require life-long commitments to self-care behaviour [72]; the results of this review suggest that elevating their self-efficacy is pivotal to support their continuous self-care behaviour. The idea of self-managing chronic disease with behavioural changes has gained traction as part of a discipline termed lifestyle medicine. Lifestyle medicine involves gaining skills and competency in adopting behaviours to promote health and addressing behaviours detrimental to health. It presents a novel approach in the treatment and prevention of non-communicable disease [73].

Psychosocial interventions to raise or maintain individuals’ self-efficacy should be incorporated into the armamentarium of treatment measures to optimise their blood pressure control. Examples of techniques healthcare providers can use to improve self-efficacy for making behavioural changes include motivational interviewing [74] and providing evaluative feedback [75]. Another intervention which healthcare providers can employ is the use of health coaches. Health coaching interventions have been shown to produce improvements in self-efficacy as well as engagement in behaviours such as undertaking physical activity and reducing dietary fat [76].

An area that requires further research is the complex relationship between self-efficacy and engagement in multiple self-care behaviours in individuals who are attempting to engage in multiple behavioural changes simultaneously. As self-efficacy is task-specific, engagement in each self-care behaviour would be driven by self-efficacy for that behaviour. Can interventions which target individuals’ self-efficacy increase self-efficacy for each of these behaviours simultaneously? Do some interventions work better at increasing self-efficacy for certain types of behaviour? This might be investigated with randomised controlled trials designed to test the effectiveness of an intervention in improving self-efficacy for multiple self-care behaviours simultaneously, or with a trial designed to test the effectiveness of various interventions in improving self-efficacy for a specific self-care behaviour.

## Conclusion

This review provides some evidence to support the association between self-efficacy and self-care behaviours in hypertension. This is consistent with Bandura’s theory that self-efficacy influences motivation and participation in self-care [13], which may in turn translate to improved health outcomes [15]. However, well-designed trials involving complex interventions are needed to prove that increasing self-efficacy will result in sustained combined self-care behaviour and favourable outcomes in essential hypertension.

## Data Availability

Data sharing is not applicable to this article as no datasets were generated or analysed during the current study.

## Abbreviations

mmHg: Millimetres of mercury
NIH: National Institute of Health
et al.: Et alia
